# Transcription of AAT•ATT Triplet Repeats in *Escherichia coli* Is Silenced by H-NS and IS1E Transposition

**DOI:** 10.1371/journal.pone.0014271

**Published:** 2010-12-09

**Authors:** Xuefeng Pan, Yuanhong Liao, Yunmeng Liu, Peng Chang, Lingni Liao, Li Yang, Hongquan Li

**Affiliations:** 1 School of Life Science, Beijing Institute of Technology, Beijing, China; 2 Institute of Cell and Molecular Biology, the University of Edinburgh, Edinburgh, United Kingdom; 3 Health Science Centre, Hebei University, Baoding, China; University of Minnesota, United States of America

## Abstract

**Background:**

The trinucleotide repeats AAT•ATT are simple DNA sequences that potentially form different types of non-B DNA secondary structures and cause genomic instabilities *in vivo*.

**Methodology and Principal Findings:**

The molecular mechanism underlying the maintenance of a 24-triplet AAT•ATT repeat was examined in *E.coli* by cloning the repeats into the *Eco*RI site in plasmid pUC18 and into the *att*B site on the *E.coli* genome. Either the AAT or the ATT strand acted as lagging strand template in a replication fork. Propagations of the repeats in either orientation on plasmids did not affect colony morphology when triplet repeat transcription using the *lacZ* promoter was repressed either by supplementing LacI^Q^ *in trans* or by adding glucose into the medium. In contrast, transparent colonies were formed by inducing transcription of the repeats, suggesting that transcription of AAT•ATT repeats was toxic to cell growth. Meanwhile, significant IS1E transposition events were observed both into the triplet repeats region proximal to the promoter side, the promoter region of the *lacZ* gene, and into the AAT•ATT region itself. Transposition reversed the transparent colony phenotype back into healthy, convex colonies. In contrast, transcription of an 8-triplet AAT•ATT repeat in either orientation on plasmids did not produce significant changes in cell morphology and did not promote IS1E transposition events. We further found that a role of IS1E transposition into plasmids was to inhibit transcription through the repeats, which was influenced by the presence of the H-NS protein, but not of its paralogue StpA.

**Conclusions and Significance:**

Our findings thus suggest that the longer AAT•ATT triplet repeats in *E.coli* become vulnerable after transcription. H-NS and its facilitated IS1E transposition can silence long triplet repeats transcription and preserve cell growth and survival.

## Introduction

AAT•TTA triplet repeats are types of trinucleotide repeats that are highly cumulative in human genome [1], [2], in contrast, they are much less abundant in many prokaryotic genomes, such as in *E.coli*, showing a biased distribution towards eukaryotic genomes [3], [4], [5], [6], [7]. Further, AAT•TTA repeats are also found to be over-represented in intronic and intergenic regions, but underrepresented in exons and UTRs, showing a nonrandom nature of distribution in an individual genome [1], [2].

AAT•TTA repeats have been characterized *in vitro* to form various types of non-B DNA secondary structures, including hairpin, triplex, non-H DNA [8], [9], [10], [11], which potentiallychallenge the stable maintenance of the repeats in genomes [8].

Consisting with this, it has been found in prokaryotes that certain proteins known as chromosome structural proteins bind preferentially to AT-rich DNA that is normally seen in promoter region, and which may make the DNA segments inactive to avoid forming non-B secondary structure during DNA transcription [12]. H-NS and its paralogue StpA are two of the main chromosome structural proteins in *E.coli*, which bind AT-rich DNA sequences with overlapped specificity; H-NS binds to more than 1000 genes when repressing the transcription of the target DNA region [12].

The non-B structures formed by AAT•TTA triplet repeats were found to be similar to those of non-B secondary structures formed by disease causing trinucleotide repeats such as CAG·CTG, CGG·CCG and GAA·TTC, which are associated with more than 40 human genetic diseases, including Huntington's disease or fragile X syndrome [8], [13]. Consistent with this, a typical expansion feature of AAT•TTA triplet repeats was found in an *in vitro* amplification assay [9], [10], [13], [14], [15], [16], [17], [18]. Moreover, in human populations similar instabilities of AAT•TTA triplet repeats were recently found to be associated with the high IgE blood syndrome in Chinese children [19], with schizophrenia [20], [21], cocaine addiction [22], and with the high prevalence of depression in adult Parkinson's disease patients [23]. More strikingly, a similar triplet repeat expansion was also found to be associated with the propagation of a 33-bp AT-rich repeat, which displayed chromosomal fragilities in humans, such as FRA3B, FRA16B, and FRA10B [24], [25].

However, the maintenance and biased distribution of AAT•TTA repeats in different chromosomal regions in a genome or in different genomes are still poorly understood. To gain some understandings of the maintenance of AAT•TTA triplet repeats *in vivo* and its biased distribution within a genome or in genomes, we performed experiments using a 24- triplet AAT•TTA repeats in the *E.coli* model system. We found that propagation of the repeats on plasmids was overall normal, but that transcription of the repeats in either orientation with either AAT or ATT serving as transcribing template induced significant changes in colony morphology, leading to formation of convex colonies from normal colonies, which then progressively transformed into transparent colonies. Interestingly, we found that IS1E transposition from the chromosome to either the proximal site of the promoter of *lac*Z in the plasmids or into the AAT•TTA triplet repeats themselves were capable of reversing colony changes by repressing transcription of the repeats. And we further found that the AT-rich repeats binding protein H-NS, a chromosomal structuring protein, but not its paralogoue StpA, played dual roles in triplet repeats binding and in facilitating IS1E transposition into the repeats containing plasmid, which ultimately prevented transcription of the triplet repeats.

## Results

### Transcription of a 24-triplet AAT•TTA repeat on a plasmid induces morphological changes of bacterial colonies from convex to transparent

Plasmids carrying a 24-triplet AAT•TTA repeat with either the AAT or the ATT strand as lagging strand template for DNA replication were initially constructed as described in the materials and methods. They were named as pAAT_24_ and pTTA_24_, respectively. Plasmid pAAT_8_ was a deletion product occasionally obtained when propagating pAAT_24_ in a *rec*A mutant, and the pTTA_8_ plasmid was obtained by reversing the AAT_8_ into the opposite orientation.

**Figure 1:**
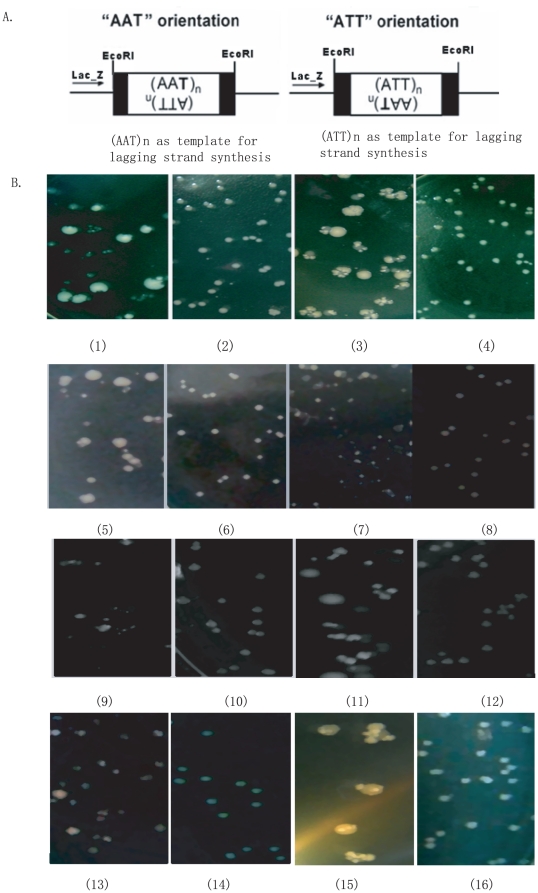
Organizations of AAT•ATT repeats in both plasmids and chromosomal *att*B site and the papillation assays when in plasmid. A) Organization of the AAT•ATT repeats in the *lac*Z gene in pUC18 plasmid and in the *att*B site of the genome; B) Papillation assays when the AAT•ATT repeats were subcloned in plasmids. (1) JM83 (pAAT_24_); (2) JM83*Δhns* (pAAT_24_); (3) JM83 (pATT_24_); (4) JM83*Δhns* (pATT_24_), which behaves similarly when a lacIQ plasmid was coexpressed, or 5% glucose was added in the LB plate (For clarity the data were not shown); (5) JM83 *ΔstpA::cat* (pAAT_24_); (6) JM83*ΔstpA::cat*(pAAT_24_) with 5% glucose; (7) JM83*ΔstpA::cat*(pATT_24_); (8) JM83*ΔstpA::cat* (pATT_24_) with 5% glucose; (9) W3110*ΔstpA::cat* (pAAT_24_); (10) W3110*ΔstpA::cat*(pAAT_24_) with 5% glucose; (11) W3110*ΔstpA::cat*(pATT_24_); (12) W3110*ΔstpA::cat*(pATT_24_) with 5% glucose; (13) AB1157 *rec*F (pAAT_24_); (14) AB1157 *rec*F (pAAT_24_) with 5% glucose; (15) AB1157 *rec*F (pATT_24_); and (16) AB1157 *rec*F (pATT_24_) with 5% glucose.

When the pAAT_24_ and pTTA_24_ plasmids were propagated in the *E.coli* JM83 wildtype strain, normal convex colonies on LB plates containing ampicillin were formed, but the colonies started to become flat when plates were left at 4°C overnight (Figure 1). Almost all flat colonies became concave, and then progressively turned into transparent colonies. In contrast, a similar phenomenon was not observed with JM83 cells when they propagated pAAT_8_, pTTA_8_, and pUC18 plasmids growing under the same conditions (data not shown). This indicated that only colonies carrying longer AAT•TTA triplet repeats suffered morphological changes when grown overnight.

Several processes may be responsible for the observed alterations in colony morphology, including triplet repeats transcription from the *lac*Z promoter (Figure 1A) [26], DNA double strand breaks caused by formation of non-B secondary structures, such as non-H structure, which may be targeted by certain DNA structure specific nucleases [27], [28], or activation of the cryptic phageΦ 80 in the JM83 chromosome, leading to cell lysis.

To test these possibilities, plasmids pAAT_24_ and pTTA_24_ were also transformed into JM83 mutants defective in homologous recombination, such as Δ*rec*A, Δ*rec*B, *rec*F::Tn10 *Kan^R^*, and Δ*ruv*ABC. We wanted to test if the colony morphology changes may be hinted at AAT•TTA triplet repeats breaks in plasmids, which requires homologous recombination repair, and which relies on the *rec* gene functions. It was observed that DNA double strand breaks in *E.coli* genome or plasmid affected cell viability and also the colony morphology, leading to plasmid loss or cell death.

However, we found that compared to wild type cells, propagations of pAAT_24_ and pTTA_24_ plasmids in all *rec* mutants tested did not significantly enhance morphological changes (data not shown). Therefore, we excluded double strand break formation as the dominant cause for the observed changes in colony morphology.

To test whether the genetic background of the *E. coli* host made a difference, the growth and colony morphology of AB1157*rec*F::Tn10 and W3110*ΔstpA::cat* cells containing plasmids pAAT_24_ and pTTA_24_ were compared to that of JM83. Although their genetic backgrounds were significantly different from JM83 and no similar cryptic phages were found, AB1157*rec*F::Tn10 and W3110*ΔstpA::cat* colonies suffered the same morphological changes as JM83 cells (Figure1 B). These suggested that the observed morphological changes as associated with AAT•TTA triplet repeats only depended on the presence of the plasmids, but not on the genetic background or cryptic phage of the host. Therefore, all subsequent work was done using the JM83 wild type and its derivative strains.

As shown in Figure 1A, AAT•ATT triplet repeats were subcloned at the *Eco*RI site of pUC18, which is located downstream from the *lac*Z promoter (Figure 1A). Therefore, we wondered whether transcription of the AAT•ATT triplet repeats from the *lac*Z promoter occurred in the stationary phase, thus causing directly or indirectly colony morphology changes.

To test whether transcription was responsible for the observed changes in colony morphology, we constructed JM83*Δhns* and JM83*Δstp*A::cat mutants defective in chromosomal structuring proteins H-NS and StpA, respectively. We therefore correlated the possible effects of transcription of AAT•ATT triplet repeats in pAAT_24_ and pTTA_24_ with morphological changes in the two strains by supplementing with IPTG, or by co-expressing LacI^Q^ from a plasmid. Interestingly, compared to JM83 and its *rec* derivatives, propagation of pAAT_24_ and pTTA_24_ in the JM83*Δhns* mutant did not provoke changes in colony morphologies, but propagation in JM83*Δstp*A::*cat* did, indicating that H-NS, but not StpA, was required for this process (Figure 1B).

H-NS binds to AT-rich DNA, which may have an effect on AAT•TTA triplet repeats transcription and somehow stimulate morphological changes of the colonies. To test additional effects, pAAT_24_ and pTTA_24_ were transformed into the Δ*hnsrec*B double mutant. No significant differences in morphological changes between the single *Δhns* and the *Δhnsrec*B double mutant were observed (data not shown). Therefore, transcription of AAT•TTA triplet repeats did not cause double strand breaks in the presence or absence of the H-NS protein.

### Transcription of AAT•ATT triplet repeats stimulates transposition of IS1E from the chromosome to plasmids

**Figure 2:**
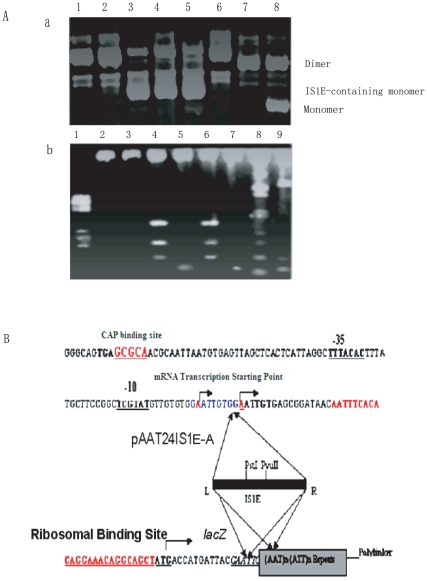
Characterization of the IS1E insertion positions. A) a. Transposition of IS1E elements into plasmids, plasmid isolated from the healthy colonies, and b. Restriction digestion of the plasmid DNA with transposons. Lanes 1, molecular weight (pBR322/BstN1); Lane 2 and 3, pAAT_24_ and pATT_24_ without IS1E transposition, digested by *Pst*I; Lane 4, pAAT_24_, with IS1E transposition, digested by *Pst*I; Lane 5, pAAT _24_ with IS1E transposition, digested by *Eco*R1;Lane 6 and 7, pAAT_24_ with IS1E transposition, digested by *Pst*I and *EcoR*I respectively; Lane 8 and 9 pAAT_24_ with IS1E transposition, digested by using *Pvu*II. B) Mapping the IS1E inserting elements in the promoter and the AAT repeats region by DNA sequencing, the positions of the mostly recognized sites were shown.

During prolonged incubation of morphologically changed colonies, secondary colonies can be slowly regenerated from the transparent cells containing pAAT_24_ and pTTA_24_ plasmids that eventually form healthy colonies *in situ* (Figure 1). The plasmid DNA was isolated from these regenerated colonies, and analyzed for variations on an agarose gel (Figure 2A). Plasmids recovered from the regenerated colonies were bigger in size, running between the monomeric and the dimeric plasmids (Figure 2A). Restriction enzyme digestions showed that extra *Pst*I cutting sites were introduced to the repeats, because pUC18 has only one *Pst*I restriction site. We sequenced the recovered plasmids and performed blast searches. This analysis indicated that the additional *Pst*I cutting sites were the result of an IS1E insertion into the plasmid.

The IS1E insertions were often on the 5′ side of the flanking sequence of the AAT•ATT triplet repeats, that is, within the promoter region of the *lac*Z gene (Figure 2B). For example, IS1E insertions occurred with a higher frequency into the “GGAATTGTG” site, where the first adenine base was the starting site for *Lac*Z mRNA transcription. Another high frequency site was “GATTACGAA”, containing the “GAA” of the *Eco*RI restriction site 5′ to the repeats (Figure 2B). These findings suggested that transcription of the AAT•ATT triplet repeats was responsible for the negative effects on cell growth.

### AAT•ATT triplet repeats serve as targets for IS1E transposition in repeats transcription

**Figure 3:**
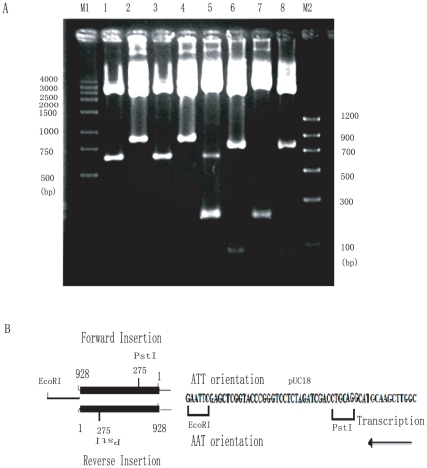
IS1E transposition into AAT•ATT repeats. A) Plasmids of AAT orientation propagated in LB medium were recovered and digested by *Pst* I (lane 1), *Eco*R I (lane 2); and propagated in LB medium in the presence of IPTG, and were digested by *Pst*I (lane 3), and *Eco*RI (lane 4); Plasmids of ATT orientation propagated in LB medium with or without IPTG, digested by *Pst* I (lane 5), *Eco*R I (lane 6); *Pst* I (lane 7 with IPTG induction) and *Eco*R I (lane 8 with IPTG induction). M1 and M2 are DNA molecular weights; B) Schematic illustration of the directions of IS1E transposition into the AAT•ATT repeats in light of the transcription of either AAT or ATT orientation.

Besides recovering IS1E insertions upstream of the AAT•ATT repeats, we also isolated plasmids containing IS1E insertions in the AAT•ATT repeating array itself (Figure 2B and Figure 3). *Eco*RI fragments of ∼1 kb in size containing at least one IS1E element within the AAT•ATT repeats were recovered from both pAAT_24_ and pTTA_24_ plasmids (Figure 3A). These findings are of particular interest, as this is the first demonstration of AAT•ATT repeats acting as potential hotspots for transposition in vivo. In this case, we have further found that the insertion of IS1E into AAT•ATT repeats rely on the repeats orientations in transcription. IS1E appeared to favour inserting itself into the AAT•ATT repeats with its unique *Pst*I restriction site distal to the *Pst*I restriction site in the vector when AAT orientation is transcribed, generating larger DNA fragment after *Pst*I digestion (Figure 3A lane 1 and 3, and 3B), while insertion of IS1E in the repeats array will use the opposite direction if the ATT orientation is transcribed, generating small DNA fragment by *Pst*I digestion (Figure 3A lane 5 and 7, and 3B). These suggested that ISIE transposition into AAT•ATT repeats occurred in repeats transcription. Transcription of AAT repeats and ATT repeats may produce distinct repeats DNA conformations that may differently be recognized by H-NS, and therefore differently affect the IS1E transpositions. Based upon the fact that IS1E transposition into AAT•ATT repeats expands the repeats by 3 triplets of either AAT or ATT repeats (9 nts) at both the 5′ and 3′ ends flanking the IS1E insertion, which may imply a potential way of making repeats expansion by certain transposons in trinucleotide repeats [13], [29], [30], [31], [32].

### Transcription of AAT•ATT triplet repeats is inactivated by IS1E transposition

To understand the biological significance of the IS1E transposition during the transcription of AAT•ATT triplet repeats, we have further analyzed the effect of IS1E transposition on *lac*Z transcription in the plasmids. To this end, we eliminated the AAT•ATT triplet repeats in pAAT_24_IS1E-A using *Eco*RI digestion (as marked in Figure 2B), and subsequently re-ligated into the repeats-free pAAT_24_IS1E-A vector at the *Eco*RI restriction site.

**Figure 4:**
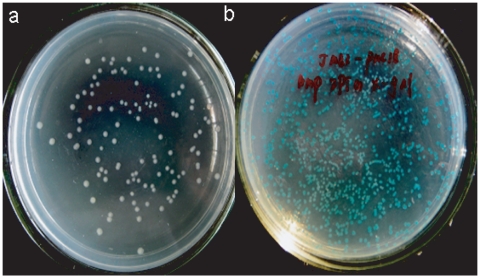
The α–complementation assay for the role of IS1E insertions. a) α-complementation by an AAT•ATT repeats free pAAT24IS1E-A plasmid, and b) α-complementation by pUC18 plasmid (For detail see text).

As determined by DNA sequencing, insertion of an IS1E into pAAT_24_IS1E-A did not alter the *lac*Z gene promoter significantly (Figure 2), however, the IS1E element could potentially use its promoter to transcribe both the transposase gene (encoded by it) and the AAT•ATT triplet repeats downstream the IS1E. We conducted a conventional α-complementation analysis by using AAT•ATT triplet repeats free pAAT_24_IS1E-A and a positive control plasmid, pUC18 by transforming these plasmids into JM83 cells. The transformants were grown on LB plates containing ampicillin, IPTG, and X-gal. JM83 cells carrying the repeats free pAAT_24_IS1E-A plasmid formed white colonies while the pUC18 plasmid formed blue colonies (Figure 4). Therefore, transposition of IS1E into the upstream region of the AAT•ATT triplet repeats in the *lac*Z promoter repressed transcription of the repeats. IS1E elements have two partially overlapping open reading frames, InsA and InsB', which have a relative reading frame of 0 and -1, respectively [29], [30], [31], [32]. InsA protein regulates transcription of the gene producing the IS1E transposease, while InsAB' binding to both the left and right IS1E terminals represses IS1E transposase transcription from a promoter found partly in IRL and simultaneously inhibits transposition [29], [30], [31], [32]. As shown here, such a regulatory mechanism may contribute to the repression of AAT•ATT triplet repeats transcription.

This has been further confirmed that repression of AAT•ATT triplet repeats transcription by IS1E insertions helped plasmid stability; we co-propagated a LacI^Q^ producing plasmid with either the pAAT_24_ or the pTTA_24_ plasmids. Co-propagation of the LacI^Q^ plasmid and pAAT_24_ or pTTA_24_ improved plasmid recovery, which was also consistent with the result of adding 5% glucose to the medium to repress *lac*Z gene transcription (data not shown). These results further indicated that transcription of AAT•ATT triplet repeats was responsible for the morphological changes of the *E.coli* cells, and that inactivation of transcription by IS1E insertions into the *lac*Z promoter or the AAT•ATT triplet repeats proximal to the promoter helped cells recover from the transcriptional stress.

### H-NS silences transcription by binding to AAT•ATT triplet repeats and promotes IS1E transposition

**Figure 5:**
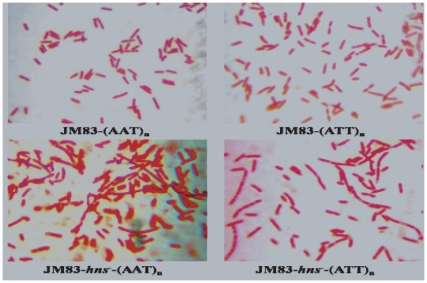
Effects of *hns* gene on the growth of AAT•ATT repeats-carrying strains in chromosome. Significant filamentous cells were observed in JM83*Δhns* -AAT and JM83*Δhns* -ATT,but cannot be seen with JM83 -AAT and JM83 -ATT, nor be seen with JM83(pAAT_24_), JM83(pATT_24_), JM83*Δhns* (pAAT_24_), JM83*Δhns*(pATT_24_) and JM83(pUC18), JM83*Δhns* (pUC18) (data not shown).

H-NS selectively silences bacterial genes associated with pathogenicity, and the gene's responses to environmental insults [33], [34], [35], [36]. H-NS binds preferentially to the AT-rich motifs displaying planar curvature, which is likely to appear in bacterial promoters [33], [36], [37]. In addition, H-NS also influences transposition and recombination [38], [39], [40]. In some transposition processes, strains with *hns* mutations show a low-level of transposase production. In IS1 transposition, reduction of InsAB' production to barely detectable levels has been reported [38], [39], [40]. Because we observed here that H-NS was required for IS1E transposition (Figure 1), while it was also implicated in the colony morphological changes in conjunction with the transcription of the 24 AAT•ATT triplet repeats, we determined whether the *hns* gene product itself may have a role in triplet repeats transcription by measuring the effects of H-NS on plasmid copy number variations in the wild type and the Δ*hns* mutant of JM83. The results indicated that transcription of the two orientations of AAT•ATT triplet repeats was differently affected by H-NS. However, the plasmid copy number of pAAT_24_ was not significantly affected by the presence or absence of H-NS, or by the presence or absence of IPTG induction (Table 1). Only 86% of the plasmid copy number of JM83 was detected in the JM83Δ*hns* mutant when induced by IPTG; 69% of the plasmid copy number of JM83 with pATT_24_ in the JM83Δ*hns* was detected without IPTG induction; and 61.7% of the plasmid copy number of JM83 was detected in JM83Δ*hns* with IPTG induction (Table 1). To rule out that multiple copies of pAAT_24_ and pTTA_24_ mitigated H-NS effects in the JM83 wild type, we integrated a single copy of a 24-triplet AAT•ATT repeat in two orientations into the *att*B site located on the *E.coli* JM83 chromosome (see Materials and Methods and Figure 1). Propagations of the repeats carrying strains were monitored by analyzing changes of bacterial cell morphology using confocal microscopy. Filamentous growth of the cells was noted when propagating the AAT•ATT repeats in the chromosome of the JM83*Δhns* mutant, but not in the JM83 wildtype (Figure 5), nor in JM83*Δhns* and JM83 strains carrying either the pAAT_24_ or pATT_24_ plasmid (data not shown). These suggested that H-NS affected the AAT•ATT triplet repeats on the chromosome in a dosage dependent manner.

**Table 1:** Effects of H-NS on the plasmid copy number.

	*JM83(AAT)*		*Δhns(AAT)*		*JM83(ATT)*			*Δhns(ATT)*	
	No IPTG	With IPTG	No IPTG	With IPTG	No IPTG	With IPTG	No IPTG		With IPTG
Plasmid/Chromo	0.592	0.586	0.620	0.553	0.568	0.536	0.396		0.316
	0.316	0.392	0.300	0.329	0.433	0.465	0.297		0.330
	0.204	0.287	0.304	0.206	0.408	0.486	0.350		0.273
Averaged Ratio	0.371	0.422	0.408	0.363	0.500	0.496	0.348		0.306

## Discussion

In human beings, the expansion and contraction of trinucleotide repeats CAG•CTG, CGG•CCG and GAA•TTC are associated with more than 40 human genetic diseases and cancers, including Fragile X, Huntington's disease, SCA1-12, MD, and Fredericha 's ataxia [8], [13]. Similarly, as one of the most abundant and most polymorphic trinucleotide repeats in the human genome, the AAT•ATT triplet repeats also show instabilities, which have recently implicated in some human health symptoms, for example, high IgE blood symptom in Chinese children [19], schizophrenia [20], [21], cocaine addiction [22], and prevalence of depression in Parkinson's disease patients [23]. However, understanding the transactions of AAT•ATT triplet repeats *in vivo* has so far been largely elusive. In this work, we found that a 24-triplet AAT•TTA repeat can be propagated stably in *E.coli* when it was not transcribed. Transcription of the triplet repeats invoked cell toxicity, and therefore had to be silenced by using H-NS or by H-NS facilitated IS1E transposition. In contrast, propagation and transcription of a short AAT•TTA repeat of 8 triplets under otherwise similar conditions did not show cell toxicity, nor H-NS and H-NS facilitated IS1E transposition, suggesting that transcription mediated cell toxicity is dependent on the length of the AAT•TTA triplet repeats, and that similar sized AAT•TTA repeat may also possibly be intrinsically vulnerable for transcription in all eukaryotes and prokaryotes. Consisting with this, we found that AAT•TTA repeats longer than 16 triplets do not exist in the databases of human genomic plus transcript, the mouse genomic plus transcript and the others as set by NCBI. Although we realized that very long AAT•TTA repeats have been found in many species, including human being and Drosophila etc., this may further implicate that transcription acted as selective pressure against long AAT•TTA repeats (>17 triplets) to be distributed in the coding region of genes.

**Figure 6:**
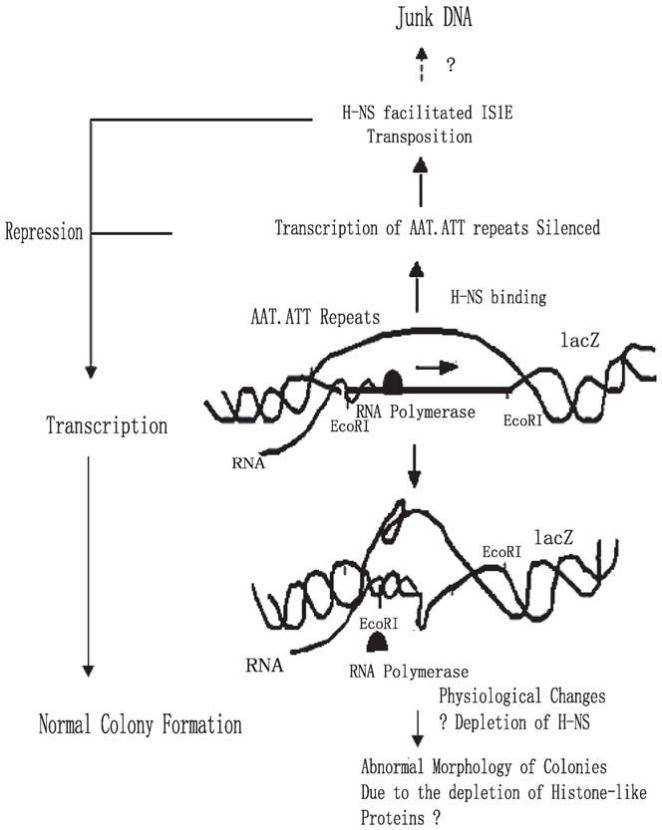
A model illustrating the response of *E.coli* cell to the transcription of AAT•ATT repeats in plasmid and genome. RNA transcription by using *lacZ* promoter opened the double stranded AAT•ATT repeats that could promote the repeats to form DNA secondary structures that may recruit histone-like proteins, H-NS, to the repeats DNA, causing depletion of this protein in the chromosome, which further resulting in DNA instability and/or cell death. Repression by H-NS deactivated the transcription while also facilitating IS1E transposion, the repeats transcription is therefore further silenced by the transposition, over time, the repeats might be able to be converted into mixed repeats by IS1E and eventually form “Junk DNA”.

The mechanism underlying the induction of cell toxicity and the cellular morphological changes by transcription of AAT•ATT triplet repeats is complex. In this work, we have ruled out the effects of generation of DNA double strand breaks and the cryptic phage induced cell lysis on colony morphological alterations. While our work directly implicated an effect of transcription of longer AAT•ATT triplet repeats on cellular morphological changes, we reasoned that transcription of longer AAT•ATT triplet repeats may facilitate the repeats to form certain types of non-B DNA secondary structures, such as non-H structure, which recruits binding of histone-like protein such as H-NS etc (Figure 6) [9], causing depletion of nuclear structure associated proteins in the chromosome in cells. The cellular morphological changes could be induced due to the depletion of the histone-like proteins, which may also includes HU and IHF etc in some situations [35], [40], [41], [42]. In support of this idea, it was found that propagation of AAT•TTA triplet repeats containing different triplets formed non-H structure in vivo [9], and also simultaneous depletions of H-NS, HU and IHF in *Escherichia coli* K-12 are lethal [41], [42]. Cells under the situation of depletion of histone-like proteins decrease the negative superhelicity of their chromosomal DNA, show increased lethality [41], [42]. Similarly, we think that might also happen during the transcription of AAT•ATT triplet repeats in plasmids that may facilitate the repeats to form non-B secondary structures, and that depletes the H-NS and affects the cell growth.

This fits with the observation of the involvement of H-NS, but not its paralogue StpA, in causing the transcription mediated problems. The H-NS was found to affect DNA compaction and transcription regulation through physical interactions with AT-rich DNA motifs with curvature [33], [34], [36], [37]. However, H-NS has also been implicated with helping targeting DNA molecules in transposition or cleaving the ends of the transposing elements (Figure 6)[30], [31], [32], [38], [39], [40]. Related to all these processes, our work suggested that H-NS affected IS1E transposition in a DNA sequence dependent manner, promoting distinct repression of AAT and ATT triplet repeats transcription and different transposition rates.

AAT•ATT triplet repeats may behave like some disease causing trinucleotide repeats, showing expansion and contraction instabilities. It was demonstrated *in vitro* assay that AAT•ATT triplet repeats produced expansion and contraction instabilities during replication [16], [17], [18]. However, similar triplet repeats instabilities were not detected in our *in vivo* assay. We have examined a large number of isogenic mutants defective in homologous recombination, methyl-directed mismatch repair, and nucleotide excision repair to search for the triplet repeats instabilities. However, under the condition of triplet repeats transcription, we failed to see any significant expansion and contraction instabilities, except for repeats instability caused by IS1E insertions (data not shown). Our work has therefore shown that longer AAT•ATT triplet repeats are vulnerable after transcription in *E.coli* cells that have therefore developed a set of mechanisms to inhibit its transcription by using both H-NS silencing and H-NS facilitated transposition inactivation, which somehow stabilize the repeats from causing cell morphological alteration and probably also repeats expansion and contraction. While bearing the findings of IS1E transposition into both the promoter region and the repeats array, we are unable to distinguish if the AAT•ATT triplet repeats serve as usual hotspots of transpositions, or does it do so only in the repeats transcription? Our findings of the IS1E transposition depended on the repeats orientation in transcription could suggest that the transposition is linked to the transcription of AAT•ATT triplet repeats. Interestingly, a similar transposition of Tn5 into GAA•TTC repeats during the repeats forming triplex has also recently been demonstrated in vitro [43].

### Conclusions

We found that transcriptions of longer AAT•ATT triplet repeats located on plasmid and in the *E.coli* genome were responsible for the formation of abnormal cell colonies. The bacterial colonies were sick in morphology, which was accompanied by significant IS1E transposition and filamentous cell growth. H-NS was found to be a key protein for these processes; presumably inactivating triplet repeats transcription by differently binding to the repeat arrays and then promoting IS1E transposition (Figure 6). These findings suggest that RNA transcription of AAT•ATT triplet repeats may serve as a selective pressure for biased distribution of AAT•ATT repeats in different chromosomal regions.

## Materials and Methods

### Bacterial strains

Bacterial strains used in this work were AB1157*rec*F:: Tn10*Kan^R^*, W3110Δ*stpA::cat*, JM83 wildtype [44], and JM83 derivatives of Δ*rec*A, *rec*B, Δ*hns*, and Δ*hnsrec*B, *ΔstpA::cat*, respectively. Strains JM83-AAT_24_ and JM83-ATT_24_, were constructed by integrating the (AAT•ATT)_ 24_ repeats at the *att*B site of the chromosome with two orientations, of which either AAT or ATT strand of the (AAT•ATT)_ 24_ served as the template for transcription. Strains JM83Δ*hns* –AAT_24_ and JM83Δ*hns* –ATT_24_ were constructed by P1 transduction of the Δ*hns* gene into the chromosome of JM83-AAT_24_, and JM83-ATT_24_, respectively. P1 transduction was performed as described in [44].

### Plasmids

The plasmids used in this study were pKOV, pUC18 and its AAT•ATT repeats carrying derivatives, pAAT_24_ and pTTA_24_ [45], [46]. Plasmid pAAT_24_ was constructed by cloning the (AAT)_24_ repeats in the *Eco*RI site of pUC18 plasmid, which was on the lagging-strand template of the replication fork (a gift from C. Abbott, University of Edinburgh). Inversion of the trinucleotide repeat array of (AAT•ATT)_24_ to generate plasmid pTTA_24_ was performed by using *Eco*RI cleavage, and followed by religation using T4 DNA ligase. The plasmids pAAT_24_ and pTTA_24_ were all confirmed by DNA sequencing. Plasmids pAAT_24_IS1E-A_,_ pAAT_24_IS1E-B, pAAT_24_IS1E-C, were plasmids of pAAT_24_ carrying IS1E elements in the promoter region of the *lac*Z (pAAT_24_IS1E-A_,_ pAAT_24_IS1E-B) or in the AAT repeat array (pAAT_24_IS1E-C), respectively. Plasmids pTTA_8_ and pTTA_8_ were the deletion products of plasmid pAAT_24_ when propagated in a JM83*recA::cat^R^* mutant. DNA sequencing primer used in this work is 5′-ATCCACATTGCCCTCCATC-3′, which was synthesized by Huada Co. Ltd (Beijing).

### Enzymes, antibiotics and biochemicals

Restriction enzymes *Eco*RI, *Pst*I, *Not*I used in this work were products of Promega (Beijing); T4 DNA ligase was purchased from New England Biolabs; Ampicillin was from Boehringer Mannheim. Isopropylthio-β-D-galactoside (IPTG) and 5-bromo-4-chloro-3-indolyl-β-D-galactopyronoside (X-gal) were bought from Sigma Chemical Company, respectively.

### Media and bacterial cultivation

Luria-Bertani broth (L-broth) was utilized for the cultivation of bacteria at 30°C and 43°C when constructing the strains of JM83-AAT_24_, JM83-ATT_24_ by using pKOV integration [44], [47]; while all other types of cultivations were carried out at 37°C. Ampicillin was applied by a concentration of 100 µg/ml when it was required. Transformation was performed by using a CaCl_2_ method [47].

### Plasmid DNA isolation and agarose gel electrophoresis

Plasmid DNA was prepared using a kit purchased from Qiagen after the propagations of the plasmid carrying strains for a period of time, normally overnight cultivation was applied. Agarose gel electrophoresis was conducted according the reference [47] on 0.8% gels (Flowgen).

### Examination for the repeat tract instability

Plasmids population was examined following the method [28]: Briefly, monomeric plasmid DNA was used to transform *E. coli* strains of interest and plasmid DNA prepared from a population of transformants. Cells from roughly 4 primary transformants were harvested in 5 ml L-broth and 50 µl of this suspension was diluted into 5 ml L-broth and grown for 24 hours. This corresponds to 30 generations of cell growth. Plasmid DNA was isolated and cleaved with *Eco*RI. The fragments were end-labelled with S^35^-dATP using DNA polymerase I Klenow fragment and resolved on 8% native polyacrylamide gels. Bands were visualised either using X-ray film or on a Molecular Dynamics phosphor Imager [28].

### DNA sequencing and determination of the transposon

DNA cycle-sequencing was performed using a kit purchased from PE Applied Biosystems. And DNA sequence was extracted from DNA sequencing has been used for searching the homology of the inserted DNA against NCBI database by using the Blast search engine on the NCBI website, IS1E transposition was therefore determined based on the Blast search.

### Determination of the plasmid copy number

Plasmid copy numbers in the strains of JM83 wildtype and JM83*Δhns* mutant carrying pAAT_24_ and pATT_24_ were determined as the following: strains carrying pAAT_24_ and pATT_24_ were initially propagated overnight in LB medium. And such overnight cultures were then made into aliquots by 1 mL into two test tubes of 1.5 mL, respectively. Induction of the repeats transcription was applied to only one sample by using IPTG by a final concentration of 0.4 mmol/L, and the two test tubes derived from the overnight culture were further cultivated for 4 hours at 37°C. Total DNA including both chromosomal DNA and plasmid DNA were prepared by following the method described in the reference [48]: Briefly, the cultures were centrifuged at 10,000 rpm at 4°C for 5 minutes. And the pellets were resuspended by adding 50 uL 10 mmol/L EDTA and 50 uL freshly prepared solution containing 0.2 moL/L, NaOH, and 0.5% sucrose, and were further incubated at 70°C for 5 min. 1.5 uL of 4 Mol/L KCl and 0.5 uL of 0.4% bromophenol blue were added when the mixture was cooled down to the room temperature, and 20 uL was analyzed by running a 0.5% agarose gel. The ratio of the total plasmid DNA and the chromosomal DNA (mainly one bulky band) was obtained by measuring their band area appeared on the agarose gel by using software (Quantity One V.4.6.2); three independent measurements were performed.

### Integration of the AAT•TTA repeats into chromosome attB site

Plasmid pKOV was used as an integrative tool for the construction of JM83-AAT_24_ and JM83-ATT_24_ [45]. Two DNA primers were designed and utilized as follows: upper strand primer: 5′ GTGTTCAGCGGCCGCTCCGGGCTATGAAATAGAAAAATGAATCCGTTGCCTGCGTTATC3′, and lower strand primer: 5′CAGGATGGCGGCCGCCCATCTGGTATCACTTAAAGGTATTAAAAACCCCACAGATGCG3′, which were synthesized by Shanghai Sangon Co. Ltd. They bear a *Not*I restriction site (underlined sequence), respectively, and also contain part of *att*B sequence, and the flanking sequence of *lac*Z open reading frame of plasmids pAAT_24_ and pTTA_24_. Integrative plasmids pKOV-AAT and pKOV-ATT were constructed by inserting the PCR products at the *Not*I sites.

Strains of JM83-AAT_24_ and JM83-ATT_24_ were selected against the following criteria [45]. For using antibiotic, 20 mg/ml of chromamphenicol was utilized; for selection against *sac*B, sucrose was added into the LB medium to a final concentration of 5%(w/v). PCR amplification using the same DNA primers as abovementioned was further performed for the confirmation of the JM83-AAT_24_, JM83-ATT_24_ .

### Observation of the cell morphological changes during cultivation

Morphological alterations of the *E.coli* vegetative cells grown in LB broth were monitored by using a light microscope with oil immersion objective. Cells were stained by safranin before observation [49].
